# Introduction to the Beckwith–Wiedemann Syndrome and Cancer Special Issue

**DOI:** 10.3390/cancers15204939

**Published:** 2023-10-11

**Authors:** Alessandro Mussa, Jennifer M. Kalish

**Affiliations:** 1 Pediatric Clinical Genetics Unit, Pediatria Specialistica, Regina Margherita Children’s Hospital, Piazza Polonia 94, 10126 Torino, Italy; 2 Department of Public Health and Pediatric Sciences, University of Torino, 10126 Torino, Italy; 3 Division of Human Genetics, Children’s Hospital of Philadelphia, Philadelphia, PA 19104, USA; 4 Center for Childhood Cancer Research, Children’s Hospital of Philadelphia, Philadelphia, PA 19104, USA; 5 Departments of Pediatrics and Genetics, Perelman School of Medicine, University of Pennsylvania, Philadelphia, PA 19104, USA

Beckwith–Wiedemann syndrome (BWS) is a genetic imprinting disorder that most commonly presents as overgrowth, macroglossia, abdominal wall defects, lateralized overgrowth, and embryonal tumors. Patients with BWS, for the most part, have a genetic or epigenetic change in chromosome 11, specifically the 11p15 region. Within BWS, there are multiple molecular subtypes that patients can have. These include loss of methylation at imprinting center 2 (IC2), gain of methylation at imprinting center 1 (IC1), paternal uniparental isodisomy, or mutations in the *CDKN1C* gene. BWS is a mosaic disorder, which means that some cells in the body may be affected while others are not. This mosaic nature of BWS implies that patients with BWS may present with a range of features. Additionally, genetic testing for BWS may be negative when a blood sample is tested because the changes are not present in the blood cells, but an affected tissue would show the genetic or epigenetic changes. The prevalence of BWS is approximately 1 in every 10,000 births. For pregnancies that are conceived using assisted reproductive technologies (ART), this prevalence increases tenfold. 

As children, patients with BWS are at higher risk for developing embryonal tumors, most commonly hepatoblastoma and Wilms tumor (nephroblastoma). Tumors develop in 2–28% of patients with BWS. Due to this increased risk, these patients are screened in childhood. Patients under the age of 3 years receive alpha-fetoprotein (AFP) screening as well as full abdominal ultrasound screening every 3 months. From ages 4 to 7, patients have renal ultrasounds every 3 months. 

This Special Issue of Cancers focuses on the link between BWS and cancer. The goal is to explore the epigenetic basis of cancer and provide insight into the biological derangements caused by the various molecular subtypes of BWS with carcinogenesis. Additional purposes of this Special Issue are to provide updated data on tumor screening in BWS patients and to give a current overview of the relationship between BWS phenotype and tumor predisposition through a variety of publications.

Tüysüz et al., in their paper “Investigation of 11p15.5 Methylation Defects Associated with Beckwith-Wiedemann Spectrum and Embryonic Tumor Risk in Lateralized Overgrowth Patients”, discussed the relationship between lateralized overgrowth and the development of embryonal tumors [1]. Methylation analysis was used to test for BWS in blood and then saliva and skin in patients with lateralized overgrowth. These patients were divided into groups based on clinical scores and genetic testing results; these groups were then followed for possible cancer development.

Luca et al., in their paper “Performance Metrics of the Scoring System for the Diagnosis of the Beckwith–Wiedemann Spectrum (BWSp) and Its Correlation with Cancer Development”, discussed the Beckwith-Wiedemann Spectrum (BWSp) Scoring System and how it is used to give a clinical score to patients with BWSp features and give them a clinical diagnosis if they have a certain score or higher [2]. In this article, a cohort of patients is examined to see the relationship between BWSp score, genetic testing results, and possible cancer development. 

Cabral de Almeida Cardoso et al., in their paper “Clinical Spectrum and Tumor Risk Analysis in Patients with Beckwith-Wiedemann Syndrome Due to CDKN1C Pathogenic Variants”, discussed one particular molecular subtype of BWS [3]. Patients with variants in the *CDKN1C* gene were examined for BWS features and given scores based on the scoring system for BWS. These patients were also followed for possible cancer development to see if there was a risk for cancer in individuals with the *CDKN1C* subtype.

Quarello et al., in their paper “Implications of an Underlying Beckwith–Wiedemann Syndrome for Wilms Tumor Treatment Strategies”, examined the risk of developing a Wilms tumor from several different cancer predisposition syndromes [4]. The connection between BWS and Wilms tumor was further investigated by looking at the characteristics and management of Wilms tumor in patients with BWS. Future treatment and monitoring guidelines were also given for the care of patients with BWS.

Soejima et al., in their paper “Placental Mesenchymal Dysplasia and Beckwith–Wiedemann Syndrome”, discussed the connection between BWS and placental mesenchymal dysplasia (PMD), and the molecular link between the two is described in detail [5]. Fetuses from PMD pregnancies are also at higher risk for hepatic tumors. The relationship between BWS, PMD, and cancer is examined in this review.

Eggermann et al., in their paper “Molecular Basis of Beckwith–Wiedemann Syndrome Spectrum with Associated Tumors and Consequences for Clinical Practice”, explored the different molecular causes of BWS and explained each in detail [6]. The overall cancer risk of BWS as well as the risk for specific tumor types are broken down and investigated based on the molecular subtypes of BWS. Treatment for the more commonly seen tumors in BWS is also discussed, and the importance of tumor screening for BWS patients is stressed. 

Cecere et al., in their paper “Co-Occurrence of Beckwith–Wiedemann Syndrome and Early-Onset Colorectal Cancer”, described a case report of a BWS patient who developed colorectal cancer at age 27 [7]. The molecular lesions were investigated at the somatic and germline levels in this patient. This article shows how, moving forward, tumor screening for patients with BWS may need to extend beyond childhood. 

Klein et al., in their paper “Occurrence of Hepatoblastomas in Patients with Beckwith-Wiedemann Spectrum (BWSp)”, discussed a case series of BWS patients who developed hepatoblastomas and examined the mosaic nature of BWS [8]. A variety of tissues were tested for each patient, including blood, liver, and tumor, if possible. This article explores the potential mechanism of hepatoblastoma development in patients with BWS and reinforces the recommendation for tumor screening for all BWS patients. 

Overall, a variety of different approaches were taken to deepen the understanding of the relationship between BWS and cancer. Typical BWS tumors were examined, as well as some that are not so commonly seen in BWS. These papers discussed BWS and cancer on a large scale through expanded cohorts and a more detail-oriented approach through case reports and molecular investigation. The articles included in this Special Issue show the need for tumor surveillance in patients with BWS and pave the way for future research in BWS and cancer. 

As often happens in medicine and biology, exceptions, unique cases, and rarities are a means to better understand general rules of biology and explore complex cellular mechanisms that are difficult to analyze in other circumstances and applicable more extensively in medicine and oncology. Decades after the discoveries regarding the complex epigenetic alterations underlying BWS, we are far from fully understanding the biological mechanisms of this unique genetic condition. At the same time, BWS still remains an inexhaustible source of biological discoveries that guide us towards a better understanding of the cellular mechanisms of carcinogenesis in general.
